# COMPARING ADVANCE DIRECTIVES LAWS IN TAIWAN AND THE UNITED STATES: IMPACT ON END-OF-LIFE CARE

**DOI:** 10.1093/geroni/igae098.2382

**Published:** 2024-12-31

**Authors:** Yufang Tu, Yuchi Young, Melissa O’Connor

**Affiliations:** North Dakota State University, Fargo, North Dakota, United States; State University of New York at Albany, Albany, New York, United States; North Dakota State University, Fargo, North Dakota, United States

## Abstract

The current study addresses increasing multicultural concerns regarding autonomy in end-of-life care decisions by comparing Advance Directives (AD) laws in the US, under the 1991 Patient Self-Determination Act (PSDA), and Taiwan, with its 2019 Patient Right to Autonomy Act (PRAA). By highlighting key legal differences and similarities, we aim to provide insights for enhancing in end-of-life care policies and understanding the impact of legal frameworks on patient autonomy internationally. To address our research objectives, we conducted a systematic literature review by searching the PubMed, ProQuest, and Hospice Foundation of Taiwan databases for the following keywords: (1) English and Chinese, (2) Published >=1991, (3) United States or Taiwan (4) Patient Self-Determination Act, Patient Right to Autonomy Act, and advance directives. The results showed that both the US and Taiwan view ADs as essential for healthcare autonomy, allowing healthcare agents to represent incapacitated individuals. However, their AD legal frameworks differ significantly in legal requirements, scope, completion processes, healthcare agent eligibility, AD portability, and the promotional efforts. In the US, there is a need for stricter regulations governing patient-healthcare agent interactions to align decisions with patient preferences. Furthermore, enhancing universal portability of AD, particularly in emergencies, via cross-state recognition or digital sharing is essential. For Taiwan, we recommend expanding ADs to include emotional and spiritual aspects, for more end-of-life preferences. Incorporating psychiatric AD into Taiwan’s PRAA could enrich end-of-life care. Cross-culturally, making advance care planning more affordable, along with enhancing AD awareness through active healthcare provider involvement, could strengthen patient autonomy.

